# Validation of the ERS standard citric acid cough challenge in healthy adult volunteers

**DOI:** 10.1186/1745-9974-6-8

**Published:** 2010-08-10

**Authors:** Caroline E Wright, Jennifer Jackson, Rachel L Thompson, Alyn H Morice

**Affiliations:** 1 Division of Cardiovascular and Respiratory Studies, Castle Hill Hospital, Cottingham, UK

## Abstract

Protocols measuring cough sensitivity can vary in terms of nebuliser, tussive agent, single and dose response. A definitive method for measuring cough sensitivity needs to be established.

The ERS guidelines recommend the KoKo DigiDoser (KD) delivery system. Study aim, was to compare the reproducibility of this citric acid (CA) cough challenge and previously established Mefar dosimeter (MD) protocol.

39 (female 26) volunteers mean age (40.4 yrs) were randomised to either KD or MD. Intra-day and inter-day reproducibility was compared.

We calculated the concentration of citric acid evoking 2 coughs (C2).

The geometric mean C2 (95%CI) was similar for both KD and MD, of 263 (200,339) mM and 209 (151,288) mM respectively.

The mean KD C2 was not significantly different. (F = 0.807, p = 0.93) from baseline over 1, 2, and 4 hrs however, the MD demonstrated significant variability (F = 7.85, P < 0.001)

Measuring mean log C2 at baseline and at 2 weeks, the KD demonstrated a stronger intraclass correlation of log C2 at baseline with 2 week log C2, ICC = 0.70 than was shown with the Mefar, ICC = 0.41

Administering CA from KD offers a reproducible cough challenge in healthy volunteers. The results correlate well with the MD challenge but offer greater intra-day and inter-day reproducibility.

**Trial Registration:**

Current controlled trials ISRCTN98385033

## Background

The methodology of citric acid cough challenge was first reported in humans over 50 yrs ago [1]. It was developed to allow for the quantification of cough reflex sensitivity and also as a tool for the assessment of antitussive therapies. Since this time many different protocols have been published and these can vary greatly in terms of the nebuliser used, the tussive agent, single breath, single dose or dose response and even the method to count number of coughs required to attain a threshold. The ERS task Force on cough methodology [2] recommended that a definitive method for measuring cough sensitivity needs to be established to allow for comparison of the results from different groups. It was suggested that the standardisation of cough challenge would lead to a higher quality of research, better drug development and ultimately improve patient care.

The tussive stimulus of citric acid has been used to demonstrate differences in cough response between the sexes [3,4] and has supported the efficacy of a number of cough medications including opiates [5] and diphenhydramine [6]. We have previously demonstrated that the most widely used antitussive, dextromethorphan, inhibited citric acid cough when given orally but not as inhalation [7]. The utility of citric acid in illustrating the pharmacokinetic and pharmacodynamic relationship of antitussives has also been demonstrated [8]. Thus citric acid is established as a tussive stimulus in cough challenge demonstrating both physiological alterations in cough reflex sensitivity as well as the pharmacological properties of antitussives.

Cough challenge methodology needs to be standardised to allow for comparison between studies, here we have compared citric acid challenge with two different methodologies and investigated the intra-day and inter-day variability to determine which provides the most reliable benchmark in clinical studies.

## Methods

### Study Subjects

40 healthy volunteers (27 female) were recruited by local advertisement. Subjects were non-smokers without symptoms of, seasonal allergy, postnasal drip or gastro-oesophageal reflux. None of the subjects had previous experience of citric acid cough challenge and were not receiving medication which might interfere with the cough reflex. All had been clear of a respiratory tract infection for 6 weeks prior to entry into the study. All volunteers gave informed consent and the study was approved by the Hull and East Riding Local Research Ethics committee.

### Study Design

Volunteers were randomly assigned to a challenge methodology. Challenges were performed by a single observer and repeated challenges were performed at the same time of day using the same allocated nebuliser pot.

Volunteers performed a baseline cough challenge on their assigned nebuliser and challenges were then repeated at 1, 2 and 4 hrs post baseline. The volunteer then returned two weeks later to perform one further challenge on the same assigned nebuliser. The challenge sequence was then repeated at a minimum of 3 days to maximum 7 days later with the alternative methodology.

### Impulse oscillometry testing

To determine the effect of citric acid cough challenge on airway tone we used Impulse oscillometry (IOS), Jaeger Masterscreen, Viasys Healthcare. IOS was performed pre and post baseline cough challenge for each of the challenge methodologies. Subjects performed 3 IOS measures each time and the mean of the 3 values was recorded. The IOS pneumotachometer was calibrated daily.

### Mefar dosimeter cough challenge

The challenge protocol was based upon our previous methodology [8]. Stock solution of citric acid 1 M (Royal Hallamshire Hospital, Sheffield) was serially diluted in physiological saline to produce incremental concentrations of citric acid (1, 3, 10, 30, 100, 300, 1000 mM). Fresh dilutions of stock solution were made on each day of testing.

The solutions were delivered to the volunteer in ascending order by a compressed air driven nebuliser controlled by a breath activated Mefar MB3 dosimeter (Mefar, Brescia, Italy). The output of the dosimeter was set at 0.1 ml/s. Each volunteer received four inhalations of each concentration of citric acid; each of one second duration, between each inhalation was a 30 second interval. The cough response for the first 15 seconds post challenge was recorded. The average of 2 or more coughs at one concentration (C2) and average of five or more coughs at one concentration (C5) were measured. The challenge was terminated once an average of five or more coughs at one concentration had occurred. Those not attaining a cough threshold at the maximum concentration were arbitrarily ascribed a C2 or C5 of 1000 mM.

### KoKo DigiDoser

Stock solution of citric acid 1 M was diluted with physiological saline to make serial doubling concentrations (7.8, 15.6, 31.2, 62.5, 125, 250, 500, 1000 mM). Fresh solutions from stock solution were made up on each day of testing.

Volunteers inhaled single breaths of citric acid from a modified 646 De Vilbiss nebuliser controlled by the KoKo DigiDoser (Pulmonary Data Service Instrumentation Inc., Louisville, CO, USA). The solutions were delivered to the volunteer in ascending order. The duration of the nebulisation was 1.2 s and nebuliser output 0.890 ml/s. Each volunteer received a single inhalation of each concentration of citric acid; between each inhalation was a 30 second interval. The cough response during the first 15 seconds of this interval was recorded. The concentration at which the volunteer coughed twice (C2) or more times and that at which volunteer coughed five (C5) or more times was recorded. The challenge was terminated once the volunteer had coughed five or more times.

## Results

### Data Analysis

All variables were tested for normal distribution and equal variance. If data not normally distributed then logarithmic transformation was used to achieve a distribution close to normality. Data was analysed used SPSS statistical software. Mean (95% confidence interval) values for IOS parameters were measured pre and post baseline challenge for the two different cough challenge methodologies.

Cough threshold values C2 and C5 were calculated by linear interpolation then log transformed. Mean cough thresholds attained from the two different methods were compared using Students *t*-test. An arbitrary level of 5% statistical significance (two tailed) was assumed.

The Bland Altman plot and 95% limits of agreement were used to examine relationship between the Mefar Dosimeter Method and KoKo method and also the deference between repeated measures on single subjects for a single method (baseline-1 hr; day 1-day 14) these were plotted against average of the 2 measurements. A Horizontal line represents the mean difference either between the two methods or the repeated measures, this should be close to Zero, limits of agreement (LOA) are plotted to define the range within which 95% of the differences between the two measurements are likely to fall.

The intra-day reproducibility of each method was analyzed by repeated measure analysis of variance (ANOVA). The intraclass correlation coefficient (ICC) was used to calculate both within-day (baseline, 1, 2 and 4 hrs) and between-day (baseline and 2-week) reliability. ICC is calculated from an analysis of variance model, It measures the amount of overall data variance due to between subject's variability (Shrout and Fleiss) [9]. The ICC can range between 0.00 (representing a totally unreliable measurement) and 1.00 (implying perfect reliability) we used a one-way random effects analysis of variance model to estimate the ICC (Donner) [10]. Other models exist (Haggard; Bartko) [11,12], but their discussion is beyond the scope of this paper. The Stata statistical computer package was used to calculate the ICC (StatCorp, 2007) [13].

### Patients

40 patients (27 female) of mean age 40 yrs (range 18-87 years) were recruited. One volunteer did not attain either a C2 or C5 and thus are not included in the analysis. Of the 39 volunteers achieving a C2 only 43% of these achieved a C5 in both methods. We therefore focus our analysis on the C2 values.

### Impulse Oscillometry

Pre and post challenge impulse oscillometry is reported in Table 1. There was no significant difference in airway resistance (R) at frequency of 5 or 20 Hz regardless of method used. There was, however, a small but significant (p < 0.05) increase in reactance (X) from -0.86 to -0.82 post challenge with the KoKo DigiDoser.

**Table 1:** IOS indices Pre and Post Citric acid Cough challenge (n = 39).

IOS Variable	CA cough challenge with KoKo DigiDoser	CA Challenge with Mefar dosimeter	P value
**Baseline**			

R5 (kPa/L/sec)	0.331 (0.30,0.36)	0.334(0.30,0.37)	0.95

R20 (kPa/L/sec)	0.281(0.26,0.31)	0.282 (0.25,0.31)	0.99

R5-R20 (kPa/L/sec)	0.050 (0.04,0.60)	0.052 (0.40,0.64)	0.89

X	-0.09 (-0.96,-0.8)	-0.09 (-0.10,-0.08)	0.77

**Response to citric acid**			

ΔR5(%)	-2.13(-5.52,1.25)	-2.51(-7.72,2.70)	0.75

ΔR20(%)	-2.27(-5.48,0.94)	-0.12(-4.30,4.08)	0.53

ΔR5-R20(%)	-5.78(-21.7,10.17)	-34.08(-68.8,0.67)	0.17

ΔX(%)	3.51(-0.64,7.65)	-6.89(-15.21,1.42)	0.03*

R5 resistance at 5 Hz; R10 resistance at 10 hz; × reactance; Δ, change in measurement after citric acid. Data represented as mean, with 95% confidence interval.

### Cough Challenge

**Figure 1:**
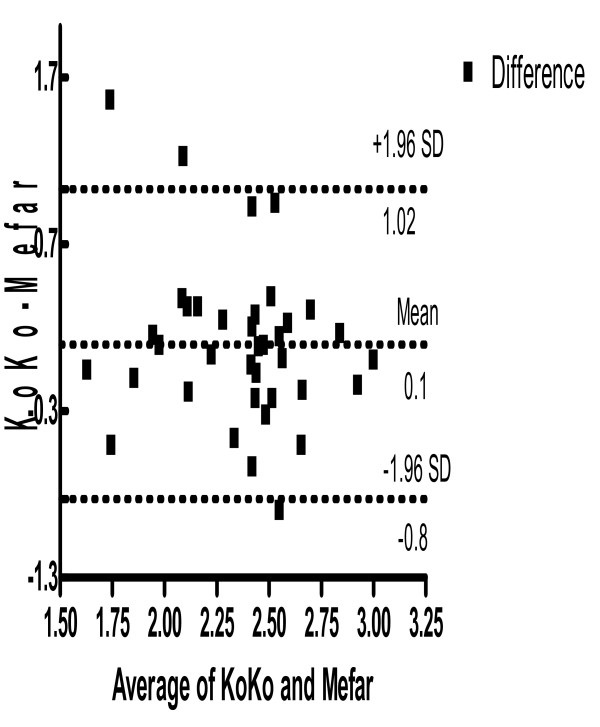
**Bland and Altman plot for cough C2 thresholds measured by KoKo DigiDoser and Mefar dosimeter Sample figure title**.

The geometric mean (range) C2 values were 263 mM (30.9-1000) for the KoKo DigiDoser and 209 log mM (9.12-1000) for the Mefar dosimeter and these values were not significantly different (Table 2). The Bland and Altman comparison of C2 is shown in Figure 1.

**Table 2:** Comparison of mean C2 measured by KoKo DigiDoser and Mefar dosimeter.

Time point	KoKo DigiDoser (n = 39)	Mefar Dosimeter (n = 39)	P value
	**Mean C2 log mM ± SD** **(95%CI)**	**Mean C2 log mM ± SD** **(95%CI)**	

Baseline	2.42 ± 0.35 (2.30-2.53)	2.32 ± 0.43 (2.18-2.46)	0.205

1 hr	2.45 ± 0.38 (2.32-2.57)	2.50 ± 0.39 (2.37-2.63)	0.455

2 hr	2.48 ± 0.37 (2.36-2.60)	2.51 ± 0.42 (2.38-2.66)	0.556

4 hr	2.45 ± 0.33 (2.34-2.55)	2.53 ± 0.41 (2.40-2.66)	0.174

2 wk	2.37 ± 0.39 (2.24-2.50)	2.45 ± 0.39 (2.32,2.58)	0.179

P value based on paired ttest

### Intra-day Repeatability

**Figure 2:**
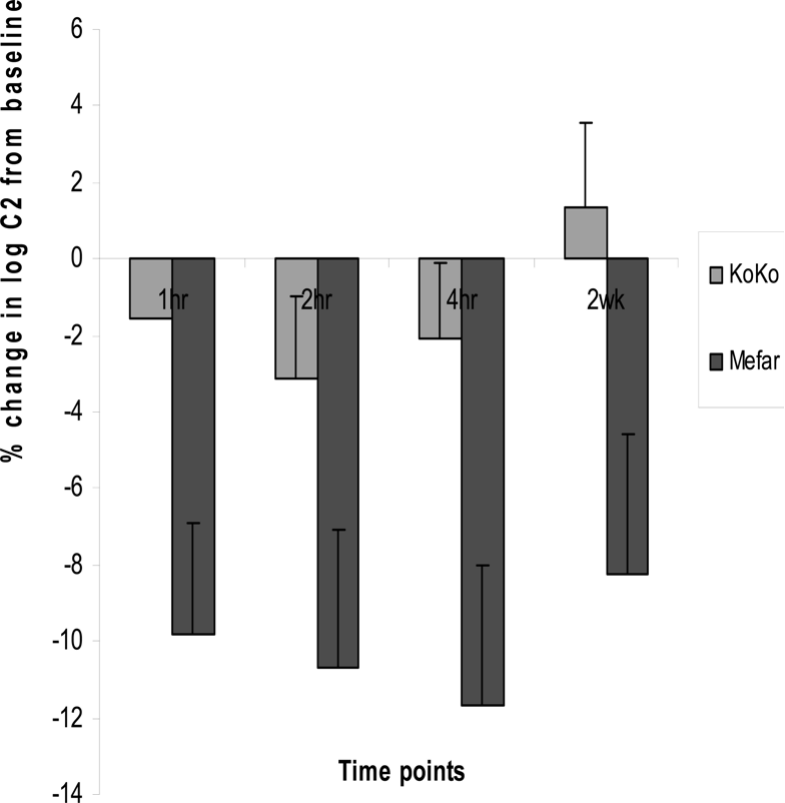
**Change in log C2 from baseline at 1,2,4 hrs and 2 wks**. Percentage change in log C2 from baseline in repeated cough challenge at 1,2,4 hrs and 2 wks comparing KoKo DigiDoser with the Mefar dosimeter

Using the KoKo DigiDoser, geometric mean C2 at baseline, and at 1, 2, and 4 hours were not significantly different F = 0.602, p = 0.61. Mean change from baseline was 1.57%, 3.14%, and 2.08% respectively (Table 3, figure 2).

**Table 3:** % Change in Mean logC2 from baseline measured at 1,2,4 hrs and 2 wks following citric acid inhalation challenge with the KoKo DigiDoser versus the Mefar Dosimeter.

Time point	KoKo DigiDoser (n = 39)	Pearson Correlation	Mefar Dosimeter (n = 39)	Pearson Correlation
			
	%Δ Mean C2 log mM (95%CI)		%ΔMean C2 log mM (95%CI)	
1 hr	-1.57 (-5.01-1.86)	0.76	-9.79 (-0.28 - -0.08)	0.74

2 hr	-3.14 (-7.50-1.23)	0.67	-10.70 (-18.0 - -3.41)	0.63

4 hr	-2.08 (-6.12-1.96)	0.75	-11.69 (-19.09 - -4.30)	0.60

2 wk	1.36 (-3.04-5.75)	0.67	-8.24 (-15.71 - -0.78)	0.56

In contrast the geometric mean C2 measured using Mefar dosimeter showed a significant increase from baseline at 1, 2, and 4 hours F = 8.91, p < 0.001. Mean change was 9.79%, 10.70%, and 11.69%, respectively (Table 3, figure 2).

C2 intra-day (baseline,1,2,4 hrs) reproducibility measured for both KoKo and Mefar showed moderate test-retest correlation coefficients of ICC = 0.68 and 0.67 respectively.

**Figure 3:**
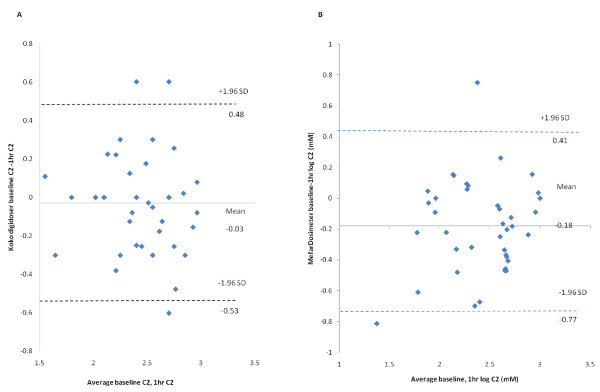
**(A) Bland Altman plot of inter-day (baseline -1 hr) repeatability of C2 measurement with KoKo Digidoser N = 39**. (B) Bland Altman plot of inter-day (baseline -1 hr) repeatability of C2 measurement with Mefar dosimeter N = 39. Intra-individual differences (n = 38) between mean log C2 measured at baseline and 1 hr post baseline plotted against the mean of the sum scores. On each plot, the central line represents the mean of the intra-individual differences, and the flanking lines represent the 95% limits of agreement.

Figure 3, Presents Bland-Altman plots of log C2 baseline and 1 hr difference scores for both the KoKo DigiDoser and Mefar dosimeter. The lOA show subject random variation between the two measurements of C2 using KoKo of 3.01 mM/-3.38 mM and with the Mefar 2.57 mM/-5.89 mM

### Inter-day repeatability

**Figure 4:**
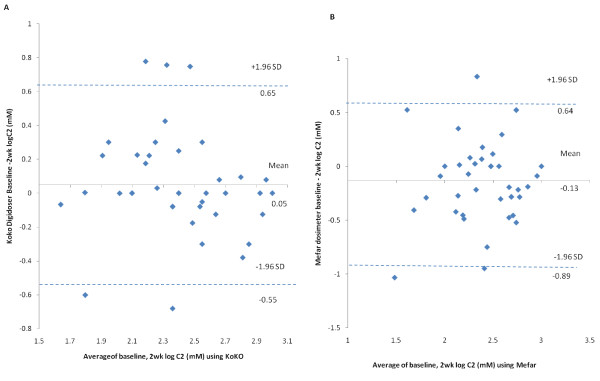
**(A) Bland Altman plot of intra-day (baseline -2 wk) repeatability of C2 measurement with KoKo DigiDoser N = 39**. (B) Bland Altman plot of intra-day (baseline -2 wk) repeatability of C2 measurement with Mefar dosimeter N = 39. Intra-individual differences (n = 38) between mean log C2 measured at baseline and 1 hr post baseline plotted against the mean of the sum scores. On each plot, the central line represents the mean of the intra-individual differences, and the flanking lines represent the 95% limits of agreement.

Intraday and inter day reproducibility of both methodologies are summarised in Table 4 and plotted in figure 3 and figure 4. The geometric mean difference in C2 between the baseline day 1 and week 2 was -0.05 mM (95% CI, 0.05 to -0.15) for the KoKo DigiDoser and 0.127 mM (95%CI, 0.25 to 0.0001) for the Mefar. The C2 measured at baseline and at 2 weeks using KoKo system showed high reproducibility, ICC = 0.70. However, ICC of C2 using mefar measured at baseline and week 2 showed low reproducibility with ICC = 0.41.

**Table 4:** Within day and between day repeatability of Mean log C2 measurements from baseline measured at 1,2,4 hrs and 2 wks following citric acid inhalation challenge with the KoKo DigiDoser versus the Mefar Dosimeter.

Method	Within day (n = 39) base 1,2,4 hrs				Between day (n = 39) baseline, 2 wks			
	
	ICC (95%CI)	SEM	CV(%)	LOA	ICC (95%CI)	SEM	CV(%)	LOA
KoKo Digidoser	0.68(0.55-0.80	0.04	-2.2	-3.38, 3.01	0.70(0.55-0.86)	0.05	4.2	-3.34, 4.46

Mefar Dosimeter	0.67(0.54-0.80)	0.05	-14.8	-5.89, 2.57	0.41(0.14-0.67)	0.06	-10.7	-7.80, 4.36

ICC: intraclass correlation, CI: confidence interval, LOA: limits of agreement, CV: coefficient of variation.

Figure 4, Presents Bland-Altman plots of log C2 baseline and 2 wk difference scores for both the KoKo DigiDoser and Mefar dosimeter. The LOA show subject random variation between the two measurements of C2 with KoKo of +4.46 mM/-3.54 mM and with the Mefar +4.36 mM/-7.80 mM.

## Discussion

Recently Dicpinigaitis [14] published a paper on the short-term and long-term reproducibility of capsaicin cough challenge testing; this study used the KoKo DigiDoser delivery system. This system uses a nebuliser which has been modified with an inspiratory flow regulator valve and the straw and baffle assembly is welded into place to optimise reproducibility.

Reproducibility of the capsaicin cough challenge studied by Dicpinigaitis demonstrated that the change in log C2 in the short term group was 0.27 ± 0.29 mM 75% of patients studied showed a within 1 doubling dose change in C2 and 95% were within 2 doubling doses. Although Dicipingatis has shown some reproducibility of the capsaicin cough challenge measures on separate days, the study was not specifically designed to determine the reproducibility of this method. Firstly there is no data on within day variability of cough challenge furthermore; the short-term group results were obtained from the collective data of patients having taken part in studies where subjects undergo cough challenge testing before and after 14 day courses of investigational drug or placebo. All cough challenges performed before and after 14 day period of placebo administration provided the short-term reproducibility data. There is some evidence in the literature that placebo treatment can significantly reduce cough [15,16] which may have affected the results of this particular study. The paper concentrated on the reproducibility of the technique but does not compare its reproducibility with that of the technique currently more commonly used, Mefar dosimeter.

This is the first study which rigorously examines and compares the reproducibility of citric acid cough challenge using two of the methodologies in common use. We have shown that the baseline measurement of C2 was highly consistent between the methods used despite considerable differences in challenge protocols and devices. This gives some confidence that results obtained in previous studies can be compared.

The C2 was measured as our endpoint in this study since, unlike capsaicin challenge it proved difficult to obtain a C5 concentration in a significant majority of normal subjects, in fact only 7 subjects achieved a C5 at all time points tested. In using C2 the mean difference between the two methods was 0.098 log, close to zero indicating the two methods are producing similar results at least on the baseline measurement, in 95% of subjects the difference between the method C2 lies between -6.7 mM to 10.7 mM. Although baseline measurements of C2 are almost identical with the two methods, repeated measurements with the KoKo DigiDoser on the same study day showed minimal subject variation of C2 between -3.38, 3.01 mM. However, In contrast when volunteers were retested with the Mefar dosimeter there was a larger reduction in C2 measured -5.89, 2.57. This was in keeping with our previous studies^8 ^and indeed was found on retesting in the original studies over 50 years ago first describing citric acid as a cough challenge agent [1]. How then does one type of inhalation challenge cause a larger reduction in subsequent C2 and the other not? In the Mefar technique a C2 is based upon the mean cough response to four inhalations of a single concentration. In each challenge the subject is therefore exposed to a much greater dose of citric acid and it may be this greater dose is responsible for the fall in cough reflex sensitivity seen. In a previous study using the Mefar technique we demonstrated cross tachyphylaxis between capsaicin and citric acid challenge. Since in man these two modalities probably act by distinct signalling pathways [17] it is possible that the act of repeated coughing itself produces down regulation. The subject coughs at least four times as much at a given concentration using the Mefar methodology.

A further possible confounding factor is that the Mefar system allows the subject to vary inspiratory flow, in previous studies variations in this can affect the citric acid cough challenge [18] and similarly also for capsaicin inhalation cough challenge [19]. A further problem with using this method is the characterization of the nebulisers, although characterized in the factory before use, the nebulisers have removable components. The nebulisers are taken apart so to allow for washing and sterilisation so when they are reattached there can be variable distances resulting between the straw and baffle assembly and the jet orifice. This can affect aerosol output and ultimately the amount of tussive agent delivered to the patient.

Another consideration is particle size. The MB3 nebulisers were shown by Praml [20] to have a particle size 5.4 μm mass median aerodynamic diameter (MMAD). Whereas Ryan [21] demonstrated 70% of particle size was of <5 μm MMAD for the Devilbiss 646 nebuliser. It could be that the larger particle size of the MB3 nebuliser may allow for a greater central airway deposition, swamping receptors and then causing down regulation.

If this theory is true we might expect an effect of citric acid on the IOS measures following the Mefar challenge. Whilst studying the cough challenge methodologies we wished to clarify whether, as it does in animals [22], citric acid administration causes significant bronchoconstriction, we used impulse oscillometry to measure any change as this has been claimed to be a more sensitive test than spirometry [23]. Surprisingly we found a significant increase in reactance as measured by Impulse oscillometry in response to citric acid inhalation delivered via the KoKo DigiDoser, this was not apparent when using the Mefar dosimeter. Respiratory reactance is defined as a composite measurement of the reciprocal of lung compliance and inertiance [24]. Reactance (X5) is numerically a negative value, and so reactance values that are less negative indicate increased compliance. Our observation that citric acid inhalation via the KoKo DigiDoser resulted in improved compliance is difficult to explain and may simply be artefact. Repeated deep breathing in normal subject's results in an increase in lung compliance [25] and our observation may reflect the respiratory manoeuvres that preceded the measurement. If a real effect then this might be due to a greater small airways delivery with the KoKo.

Somewhat against the hypothesis that the dose and delivery of the Mefar challenge are the cause of the diminished response is that, even at two weeks post initial challenge, our volunteers still had a cough response which was diminished by 8%. Again the KoKo challenge was unaltered, the cough sensitivity being slightly enhanced by 1.4%. The most likely explanation is that after the initial inhalation using the Mefar the volunteer is aware of the stimulus and that the challenge can become unpleasant at higher concentrations of citric acid thus the volunteer through a learned response, on subsequent inhalations may not inhale as strongly as they did at first, this is possible with the Mefar as the flow through the nebuliser is unregulated unlike the KoKo DigiDoser where flow through the nebuliser is regulated such that flow is limited.

In truth we have no cogent explanation why, as in other observations [1,8] there is sustained damping down of cough reflex sensitivity to citric acid with the Mefar technique and why this is not seen with the KoKo DigiDoser. However knowledge of this is clearly of great importance in the design and analysis of pharmaceutical studies since we are often looking at a small therapeutic effect of antitussives. We have previously shown "placebo" effects of 8% and drug effect 25% [26,27]. It is quite possible that the placebo may have had little or no effect, and was simply an artefact of repeated citric acid challenge.

The artificial induction of cough is an important methodology in cough research. It is useful in establishing the relation between pathology and cough sensitivity and it is an invaluable objective measure of cough sensitivity in relation to pharmacological intervention. As in any methodology it is important to have reproducible and sensitive measures free of artefact. We have confirmed the recommendations of the ERS task force on standardizing a method for performance of cough challenges and this should go a long way to facilitate use of the DigiDoser methodology across different laboratories, and allow for universal interpretation and comparison of data the KoKo DigiDoser is to be the method of choice being highly reproducible in the short and medium term typically used in pharmaceutical studies.

## Competing interests

The authors declare that they have no competing interests.

## Authors' contributions

JJ carried out the coordination and completion of the study and performed cough Challenge and spirometry on the majority of volunteers. RT participated in the design of the study and some patient testing. AHM conceived the study, and helped to draft the manuscript. CW designed the performed the statistical analysis and drafted the manuscript. All authors read and approved the final manuscript.
